# Immunomodulatory effects of mesenchymal stem cells in peripheral nerve injury

**DOI:** 10.1186/s13287-021-02690-2

**Published:** 2022-01-15

**Authors:** Xiangling Li, Yanjun Guan, Chaochao Li, Tieyuan Zhang, Fanqi Meng, Jian Zhang, Junyang Li, Shengfeng Chen, Qi Wang, Yi Wang, Jiang Peng, Jinshu Tang

**Affiliations:** 1 The Fourth Medical Center of Chinese PLA General Hospital, Beijing, 100853 People’s Republic of China; 2 Institute of Orthopedics, Chinese PLA General Hospital, Beijing Key Lab of Regenerative Medicine in Orthopedics, Key Laboratory of Musculoskeletal Trauma & War Injuries PLA, Beijing, 100853 People’s Republic of China; 3 Department of Stomatology, First Medical Center, Chinese PLA General Hospital, Beijing, 100853 People’s Republic of China; 4 The School of Medicine, Jinzhou Medical University, Jinzhou, 121099 People’s Republic of China; 5 Department of Spine Surgery, Peking University People’s Hospital, Beijing, 100044 People’s Republic of China; 6 The School of Medicine, Nankai University, Tianjin, 300071 People’s Republic of China

**Keywords:** Mesenchymal stem cells (MSCs), Immunomodulation, Peripheral nerve injury, Review

## Abstract

Various immune cells and cytokines are present in the aftermath of peripheral nerve injuries (PNI), and coordination of the local inflammatory response is of great significance for the recovery of PNI. Mesenchymal stem cells (MSCs) exhibit immunosuppressive and anti-inflammatory abilities which can accelerate tissue regeneration and attenuate inflammation, but the role of MSCs in the regulation of the local inflammatory microenvironment after PNI has not been widely studied. Here, we summarize the known interactions between MSCs, immune cells, and inflammatory cytokines following PNI with a focus on the immunosuppressive role of MSCs. We also discuss the immunomodulatory potential of MSC-derived extracellular vesicles as a new cell-free treatment for PNI.

## Background

Peripheral nerve injury (PNI) refers to varying degrees of trauma to peripheral nerve stems or branches. PNI accounts for 1.5–4.0% of global trauma cases annually [1] and is one of the most challenging health issues at present. Nerve regeneration is a complicated cellular process involving inflammation, neurotrophic factors, neurotransmitters, adhesion, the formation of axons and growth cones, and the survival of neurons [2]. PNI triggers a series of immunoregulatory reactions in the cellular microenvironment, involving changes of immune cells and related immunoregulatory factors. Immunomodulatory factors currently known include interleukin-1 (IL-1α, IL-1β), IL-2, IL-4, IL-6, IL-10, IL-12, IL-13, IL-17, IL-6 [3, 4], tumor necrosis factor-a (TNF-α), indoleamine-2, 3-dioxygenase (IDO), interferon-γ (IFN-γ), transforming growth factor-beta1 (TGF-β1), heme oxygenase-1 (HO-1), hepatocyte growth factor (HGF), nitricoxide (NO), human leucocyte antigen-G5 (HLA-G5) and prostaglandin E2 (PGE2) [5, 6].

**Fig. 1:**
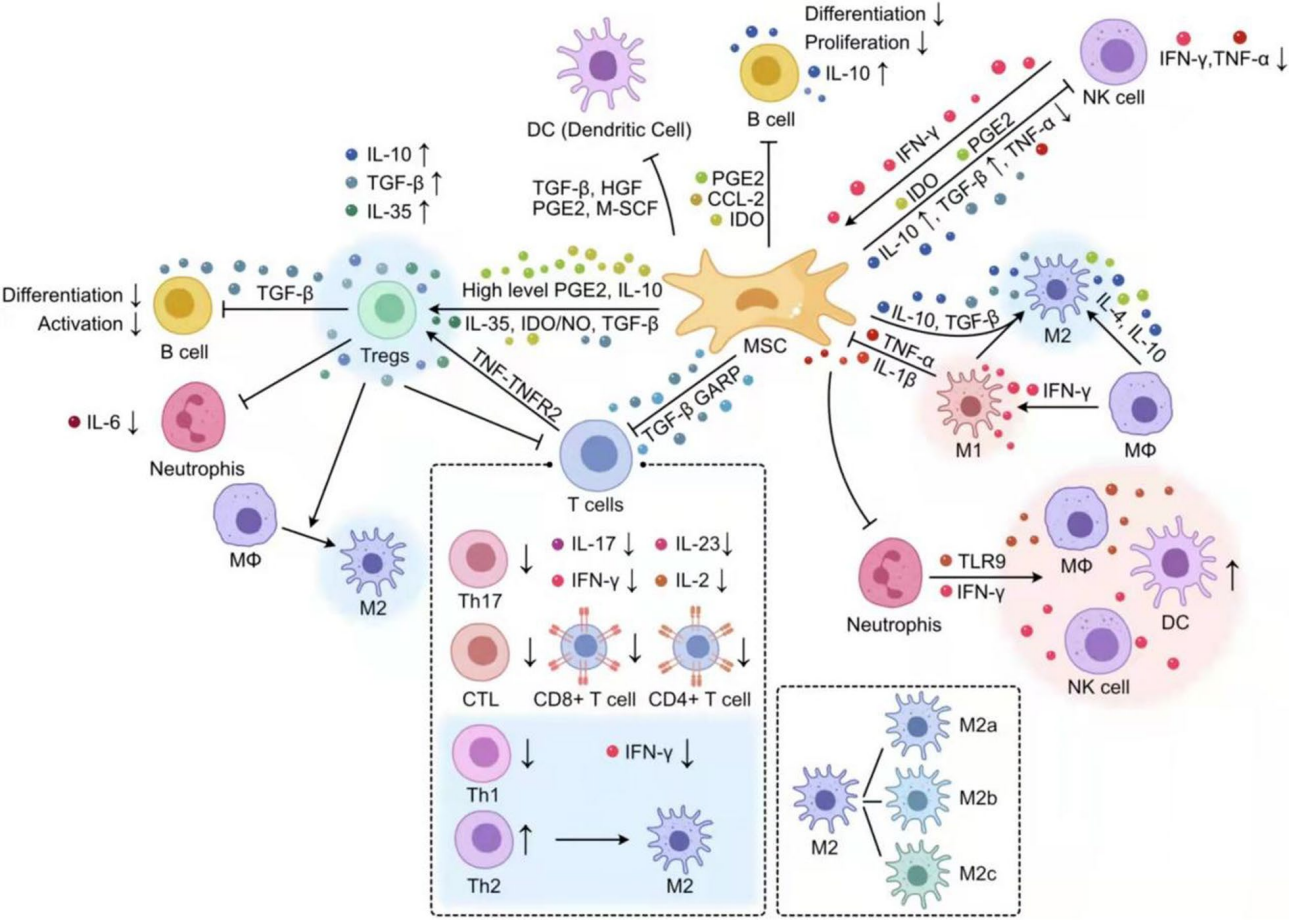
Paracrine immunomodulatory effects of MSCs on different immune cells. MSCs exhibited immunosuppressive effects on macrophages, T-cells, B-cells, regulatory T-cells (Tregs), DCs, neutrophils, and NK cells through the secretion of various cytokines. MSCs could inhibit macrophage migration and promoted a shift from M1 to M2 polarization

Amongst other techniques, autologous nerve grafting, nerve conduit synthesis [7], stem cell transplantation [8], and exosome extraction [9] are currently more popular treatments for PNI. For peripheral nerve damage of more than 3 cm in length, autologous nerve grafting is considered the gold standard treatment [10]. Stem cell transplantation is a novel method capable of regulating the inflammatory response which may accelerate the transition from destructive to restorative inflammatory microenvironment [11] and has been confirmed to promote the regeneration of peripheral and central nerves [12]. Mesenchymal stem cells (MSCs) can produce a variety of immunoregulatory factors modulating the immune function of autologous and allogeneic immune cells as well as innate (including natural killer (NK) cells[13], neutrophils, macrophages, mast cells, and dendritic cells (DCs)) and acquired immune cells (including T cells and B cells) [14] (Fig. 1). MSCs exert their immunomodulatory role via two distinct mechanisms: secretion of cytokines, including IDO (human) or NO (mouse), PGE2, IL-4, IL-10, IL-12, and IFN-γ, and TNF-α, in a paracrine manner, and direct contact between cells [15]. The most important role of human MSCs (hMSCs) following injury is their secretion of bioactive molecules such as cytokines, chemokines, and growth factors, rather than their differentiation ability [16] and there are now several studies that have shown that many of the beneficial effects attributed to stem cell therapy may be mediated via paracrine mechanisms [17]. However, the immunomodulatory effects exerted by MSCs after PNI are not entirely clear. In this review, we discuss the changes occurring in the inflammatory microenvironment after treatment of PNI with MSCs and MSC-derived exosomes (exos), as well as the immunomodulatory effects exerted by different immune cells and inflammatory factors.

## MSCs exert immunomodulatory effects by modulating cytokine expression

### MSCs downregulate the expression of pro-inflammatory factors

It has been reported that MSCs can suppress the expression of a variety of inflammatory cytokines in different diseases, such as IL-17, IL-1β, IL-6, IL-3, IL-8, IL-21, IL-22, IL-7, IFN-γ, TNF-α, and IL-12, among others. After PNI, pro-inflammatory factors such as IL-1, IL-2, IL-6, and TNF-α are expressed during the first stage of Wallerian degeneration, which promotes the recruitment of macrophages within 2–3 days [18]. However, the inflammatory response exerts different functions at different timepoints following injury. Results by Mietto et al. suggested that in the early stage of injury, proinflammatory cytokines such as IL-1β and TNF-α are predominantly secreted by de-differentiated Schwann cells (SCs) after losing contact with axons, which then recruit macrophages to the site of injury [19]. Chen et al. found that pro-inflammatory cytokines surrounding the injured peripheral nerve were significantly increased in the lymph nodes (LN), creating a pro-inflammatory environment, thereby leading to autoimmune reactions against peripheral nerve antigens [20]. MSCs exhibit strong plasticity and can switch from the pro-inflammatory “MSC1” type to the anti-inflammatory “MSC2” type. Following stimulation with pro-inflammatory factors such as IL-1α, TNF-α, and IFN-γ, or after toll like receptor 3 (TLR3) activation, resting-state MSCs are polarized to the anti-inflammatory MSC2 type, producing large amounts of NO/IDO and other immunoregulatory factors and chemokines which play an immunosuppressive role by inhibiting the function of effector T cells and inducing the production of regulatory T cells (Treg cells) [14]. Moreover, Omi [21] et al. reported that DPSC transplantation can significantly reduce the expression of pro-inflammatory cytokines IL-1β and TNF-α and increase M2 polarization and recruitment under the inflammatory condition of diabetic peripheral nerves. In addition, among the numerous proinflammatory factors affected by MSCs after PNI, IFN-γ, IL-1, TNF-α, IL-6, and IL-17 have been widely studied in peripheral nerve regeneration. Below is some simple summary of them.

#### MSCs suppress the expression of IFN-γ

Previous studies have shown that low levels of IFN-γ can accelerate the spread of stem cells. SCs exhibited higher levels of proliferation and expression of elevated GDNF levels when cultured in a medium containing IFN-γ [22]. Several studies have also shown that MSCs can suppress the expression of IFN-γ. For instance, Aggarwal et al. found that co-culture of different immune cells with hMSCs resulted in an inhibition of IFN-γ expression and an increase in IL-4 secretion [23], increased the transition of pro-inflammatory Th1 cells to anti-inflammatory Th2 cells, and facilitated the generation of CD25 + and CD4 + regulatory T cells. Other studies have proven that IFN-γ can enhance the immunomodulatory capacity of MSCs. For instance, Kim [24] et al. reported that IFN-γ-primed MSCs could not only upregulate the gene expression of chemotactic factors cysteine-X-cysteine chemokine ligand 9 (CXCL9), CXCL10, CC-chemokine ligand 8 (CCL8), which may possess a significant potential to recruit leukocytes but also highly promote the IDO expression through the JAK-STAT1 signaling pathway, thereby contributing to the inhibition of T-cells mediated by MSCs. Domenis et al. suggested that the pro-inflammatory cytokines IFN-γ/TNF-α may improve the immunomodulatory and anti-inflammatory potential of exosomes released by adipose-derived mesenchymal stem cells (AD-MSCs) promote macrophage polarization toward the M2 type, and the authors also speculated that the immunomodulatory properties of AD-MSCs-derived exosomes may be a result of the inflammatory microenvironment [25].

#### MSCs decrease the expression of IL-1

There are two types of IL-1, IL-1α, IL-1β. The basic function of IL-1α is to induce the generation of IL-6 and granulocyte–macrophage colony-stimulating factor (GM-CSF) in fibroblasts in situ, which can be detected within 2 to 5 h after an injury [26]. IL-1β may promote nerve regeneration via the nuclear factor-κB (NF-κB) signaling pathway [27]. Previous studies suggested that there are low levels of IL-1β one hour after injury, but one-day post-injury—before macrophage recruitment—the secretion of IL-1β peaks [28], 6 and 24 h after injury, the expression levels of IL-1β is 2 times and 10 times higher than in the control groups [29, 30]. High levels of IL-1β are maintained for several days [27], gradually decreasing and finally returning to the baseline levels around 14 days after PNI. MSCs can reduce IL-1 expression: for instance, a study by Chen et al. reported that bone-marrow-derived MSCs significantly reduced the expression of IL-1β in mice following spinal cord injury (SCI) [31].

#### MSCs downregulate the secretion of TNF-α

TNF can be regenerated by monocytes, macrophages, and other immune cells such as DCs, B cells, activated NK cells, and T cells [32]. Similar to IL-1β, TNF-α reaches its secretory peak one day after PNI [28] In addition, TNF can promote nerve regeneration by controlling the number of neutrophils and IL-1β expression [33]. The proinflammatory microenvironment mediated by TNF-α plays a significant role in the immunoregulatory function of MSCs. For example, pre-treatment of MSCs with TNF-α promotes the secretion of anti-inflammatory cytokines including IL-10 and TGF-β, enhancing immunosuppression and inducing anti-inflammatory Tregs [34]. Inhibition of the TNF-α-TNFR2 signaling pathway in MSCs caused a decreased IL-10 and TGF-β secretion, while increasing secretion of TNF-α, INF-γ, IL-2, and IL-17 by effector T cells (Teffs) [35]. The protective functions mediated via the TNFα-TNFR2 pathway result in cell proliferation and survival [36]. However, TNF-α has also been shown to inhibit the function of MSCs in nerve repair by activating the Wnt signaling pathway under certain circumstances [37].

#### MSCs reduce the expression of IL-6

IL-6 is a pluripotent cytokine that not only can accelerate the proliferation and activation of effector T cells through immunostimulation, but also regulate Treg cells through immunosuppression [26, 38]. IL-6 is not detected in intact nerves, but recent studies have revealed that the cytokine IL-6 is produced by macrophages and fibroblasts in the injured peripheral nerve [39], and its expression is regulated by TNF-α. After sciatic nerve injury, macrophage-derived IL-6 levels are increased within 2 h. In SCs, its expression is increased within 3 h of injury and maintained for at least 21 days. Co-culturing the facial nerve with BMSCs in vitro*,* Ge et al. and found that BMSCs secrete high levels of IL-6 to regulate the balance of CD4 + T cell subsets, to achieve future treatment of facial nerve injury [40]. Baez-Jurado demonstrated that astrocytes co-cultured with the conditioned medium from AD-MSCs reduced the expression levels of IL-6, TNF-α, and GM-CSF and increased the expression levels of neuroprotective cytokines [41] to regulate brain inflammation. Moreover, GM-CSF has been shown to suppress inflammation and potentially improve the microenvironment toward promoting the clearance of myelin debris by increasing the expression of galectin-3 on SCs [42] and enabling axonal regeneration [43]. GM-CSF plays a key role in the development and maturation of DCs as well as in the proliferation and activation of T-cells [44].

#### MSCs downregulate the expression of IL-17

IL-17 is produced by activated CD4 + T cells and increases the production of proinflammatory factors and neutrophil mobilizing cytokines, such as TNF-α, IL-1β, and G-CSF [45]. IL-17 has been shown to play a crucial role in the regulation of inflammatory responses in autoimmune diseases. IL-17 can promote the release of inflammatory factors TNF-α, IL-6, and IFN-γ [46]. IL-15 and IL-23, derived from dendritic cells and macrophages, respectively, are the most significant regulatory factors of the expression of IL-17A [47]. The expression of these two factors increases on the first day after sciatic nerve injury, and their peaks precede the peak expression of IL-17A, suggesting that they act as inducers of IL-17A after PNI. The expression of IL-17A peaks on day 7 after PNI [48]. The infiltration of inflammatory cells and levels of pro-inflammatory cytokines surrounding the injury site were significantly reduced in IL-17-knockout mice compared to controls after partial sciatic nerve ligation was significantly reduced, while the expression of anti-inflammatory cytokines IL-10, IL-13, and enkephalin were increased. Yining et al. found that BMSCs can promote the proliferation and differentiation of Th17 cells which secrete IL-17 and in turn promote BMSC secretion of PGE2, while high levels of PGE2 negatively regulate IL-17 secretion by Th17 cells [40].

### MSCs exert immunomodulatory effects after PNI by increasing the expression of anti-inflammatory factors

Following SCI, the anti-inflammatory factors IL-10 and TGF-β can be detected after PNI following macrophage recruitment [18], which can reduce the inflammatory response and promote neural tissue regeneration [49]. The release of IL-4 and IL-13 by Th2 can accelerate the activation of M2 macrophages, promote the expression of anti-inflammatory cytokines IL-10 and TGF-β, and up-regulate the expression of extracellular matrix protein, growth factor, and arginase [50]. Several studies have revealed that MSCs can promote the expression of anti-inflammatory factors. For instance, Aline et al. found that mice treated with Wharton’s jelly-derived MSCs (WJ-MSCs) exhibited increased levels of IL-4, IL-10, IL-35, and TGF-β [51]. The authors concluded hWJ-MSCs may regulate immune cell function via secretion of high levels of TGF-β and IL-35, to adjust the inflammatory microenvironment to promote the recovery of the sciatic nerve. In this review, we have focused on describing the main representative anti-inflammatory factors IL-10 and IL-4. However, it should be noted that IL-4 and IL-10 are not the sole representatives after PNI.

#### MSCs increase the expression of IL-10

IL-10 is a type of pro-regenerative cytokine. Increased expression of IL-10 is associated with wound healing, tissue remodeling, and myelin regeneration [51]. It has been suggested that the production and secretion of IL-10 are induced by fibroblasts within 5 h after injury, but the low levels of IL-10 produced by fibroblasts are insufficient to aid substantial repair. To overcome this, recruited macrophages, who are the key cell type expressing IL-10, produce and secrete IL-10. mRNA levels of IL-10 increase from the first day after the injury, increase significantly on day 4, peak on day 7, and are maintained at high levels until day 14 [18, 53]. Protein expression of IL-10 gradually increases in the first two weeks after injury [53]. IL-10 can limit the expression of proinflammatory cytokines IL-1, TNF-α, and IL-6, as well as Th1-derived cytokines such as IL-2 and IFN-γ. Moreover, IL-10 regulates the differentiation and proliferation of macrophages, T cells, and B cells [26]. Ydens et al. found that sciatic nerve transection injury can induce an immunosuppressive response, increase levels of IL-10, and provide a microenvironment conducive to macrophage polarization to M2 type [54]. Additionally, Obermajer et al. found that both IL-10 and HLA-G5 are essential for MSC-mediated immunosuppression, and the two molecules exert synergistic effects[55], and can also enhance the anti-inflammatory potential of MSCs. Cui et al. showed that BMSCs stimulated with IL-10 inhibit the expression of TNF-α and IL-1β [56].

#### MSCs promote the expression of IL-4

It is becoming increasingly clear that IL-4 is an important immunomodulatory cytokine. It can not only recruit more macrophages but also promote macrophage M2 type polarization, which has the potential to promote peripheral nerve regeneration [57]. MSCs expressing IL-4 can induce macrophage M2 polarization [11] and further promote the expression of IL-4. In addition, M2 macrophages induced by IL-4 or the other anti-inflammatory cytokines can suppress anti-inflammatory responses and promote angiogenesis and axonal regeneration [58]. Ullah et al. found that the expression IL-4 and TGF-β were reduced at 2 weeks after sciatic nerve injury when treated with dental pulp-derived stem cells (DPSCs), whose levels gradually increased at 8 and 12 weeks after injury [59]. Moreover, Yagura et al. demonstrated that transplanted MSCs could induce euronal cells to secrete CCL5 binding to the macrophage surface, which induced high levels of IL-4 expression to promote macrophage polarization to type M2 [60].

## Immunomodulatory effects of MSCs on immune cells in PNI

### MSCs promote macrophage polarization toward M2 type

During acute inflammation, macrophages can amplify the inflammatory response and recruit additional immune cells via phagocytosis of invading microorganisms [61]. Two to three days after an injury, blood-derived macrophages in the distal nerve begin to accumulate and reach their numbers peak around 7-14d after injury. Macrophages infiltrating the injured nerve express and produce a great number of cytokines such as CCL2 and TNF-α, IL-1α, and IL-1β, to promote the further recruitment of monocytes or macrophages [28, 62]. Both cell-to-cell contact and secreted factors are essential for the modulation of macrophages by MSCs. It has been confirmed that MSCs can secrete a variety of growth factors, chemokines, as well as other signaling molecules to regulate macrophage maturation, polarization, and proliferation [63, 64]. For instance, Zhong et al. reported that BMSCs can secrete GDNF that converts microglial destructive M1-phenotype into regenerative M2-phenotype for the suppression of neuroinflammation, possibly through the inhibition of NF-κB signaling pathway and the promotion of the PI3K/AKT signaling pathway [65]. Additionally, several studies have shown that MSCs can influence M1/M2 polarization. For example, by co-culturing ADMSCs with human peripheral blood monocytes (PBMCs) to explore the effects of MSC-induced macrophages (iMΦ) on inflammation modulation, Heo et al. found that macrophages highly expressed M2 type markers, indicating that secreted factors derived from MSCs promoted M2 polarization [66]. Using co-culture of bone marrow-derived macrophages with MSCs, Xu et al. found that MSCs significantly promoted macrophage polarization from M1 to M2 [67]. MSCs are thought to modulate activation and polarization of macrophages via four molecular pathways which down-regulate the release of pro-inflammatory cytokines and accelerate nerve repair and regeneration, including PGE2, tumor-necrosis-factor-inducible gene 6 protein (TSG-6), progesterone receptor (PR), and glucocorticoid receptors (GR). Chen et al. found that exosomes derived from human umbilical cord-derived MSCs (hUC-MSCs) could modulate macrophages towards M2 polarization [68]. Peruzzaro et al. showed that MSCs can change the ratio of classical to M2 macrophage/microglia phenotypes following traumatic brain injuries (TBI), increasing the proportion of M2 cells around the injury site [64]. Moreover, macrophages can also influence MSCs. For example, Ma et al. found that macrophages can inhibit the proliferation and differentiation of ADSCs by secreting pro-inflammatory cytokines, mainly TNF-α and IL-1β [69]. Several studies have shown that NF-κB is a crucial regulator of inflammatory signaling and molecular determinant of macrophage phenotype by inducing inflammatory mediators such as IL-1β, IL-6, TNF, IL-12, CXCL-8, and cyclooxygenase (COX) [70]. John et al. proposed that the inhibition of NF-κB signaling macrophages could activate neutrophils as a result of the reduced release of proinflammatory cytokines [71].

In conclusion, MSCs can decrease the infiltration of M1 macrophages and promote macrophage polarization from M1 to M2 type, thereby reducing pro-inflammatory and increasing anti-inflammatory cytokines.

#### MSCs reduce the expression of T lymphocytes

Similar to macrophages, T lymphocytes are essential for peripheral nerve regeneration. T cells can regulate nerve regeneration through a variety of mechanisms, including the secretion of cytokines, release of growth factors, or direct interaction with cells [72]. MSCs can suppress the proliferation of activated T lymphocytes in a dose-dependent manner, which is thought to be mediated by the expression of IDO and COX2 [73]. MSCs have been proven to exhibit immunomodulatory effects on T cells in vitro. For instance, MSC-conditioned medium promotes T-cell production of anti-inflammatory cytokines TGF-β, IL-4, IL-10, NO, and IDO [74]. Heo et al. found that iMΦ strongly inhibited T lymphocytes [66]. Furthermore, MSC also has been shown to exert immunosuppressive functions on T cells in vivo. Siniscalco et al. found that hMSCs delivered systemically via the tail vein decrease secretion levels of IL-1β and IL-17 and increase expression levels of IL-10 and the CD206 macrophage marker in a mouse model of neuropathic pain, thus the authors concluded that T lymphocytes are associated with the production of pro-inflammatory cytokines IL-17 and IL-1β [75]. Alternatively, although T cells do not produce IL-4, they have been shown to recruit eosinophils that can express IL-4, both in vivo and in vitro, to promote nerve regeneration [76]. MSCs can inhibit T cell proliferation by promoting the multiplication of Tregs [77], and suppressing conventional T cells, converting them to Tregs [78]. Moreover, umbilical cord blood-derived MSCs pretreated with IFN-γ could suppress the function of mature dendritic cells, thus stimulating T-lymphocyte proliferation after direct contact [79].

In summary, MSCs could reduce the inflammatory response by inhibiting T-cells proliferation and activation.

#### MSCs promote the proliferation and differentiation of Treg cells

MSCs transplantation promotes the expansion of Tregs in injured tissues [80]. Maintaining the balance between Tregs and inflammatory cytokines is an important immunoregulatory role of MSCs. For instance, several studies found that IL-6 combined with TGF-β can induce Th17 cell differentiation, while TGF-β induces differentiation of Tregs [62]. Tregs produce proinflammatory factors under certain conditions, but generally produce anti-inflammatory cytokines such as IL-10, TGF-β, and IL-35, which participate in the immunosuppressive function of Tregs, promoting functional recovery of injured peripheral nerve [81, 82]. TNF can not only reduce the suppressive function of Treg cells, but also promote their proliferation under certain conditions in vitro [83]. Aline et al. found that hWJ-MSC promotes higher expression levels of anti-inflammatory cytokines IL-35 and TGF-β in Tregs when compared with that in fibroblasts [51]. In the treatment of PNI, an increased level of IL-35 can induce Tregs differentiation [84]. In addition interacting with cytokines, Tregs can induce neutrophil apoptosis, promote the expression of TGF-β1 and IL-10, and inhibit neutrophil production of IL-6 to create anti-inflammatory conditions that favor tissue repair. Additionally, IL-10 also can counteract Tregs [85]. Tregs contribute to the inhibition of monocyte secretion of inflammatory factors that promote macrophage polarization toward the M2 phenotype.

Therefore, MSCs can induce the generation of Tregs in the inflammatory microenvironment so as to inhibit the immune response and attenuate the resultant inflammation.

#### MSCs inhibit the activation of NK cells

Several studies have shown that MSCs may inhibit NK cell proliferation and cytotoxic activity [86], including suppression of proliferation of resting NK cells induced by IL-2, the secretion of pro-inflammatory factors such as TNF-α and IFN-γ, and their killing capacity on target cells. The inhibitory effect of MSCs on NK cells is dose-dependent and a significant inhibitory effect can only be achieved by maintaining a high MSC/NK cells [87]. Noone et al. proposed that hUCMSCs can inhibit NK cell activation via secretion of PGE2 [13]. Aggarwal et al. found a statistically significant reduction in IFN-γ production after co-culture of hMSCs with IL-2-stimulated NK cells [23]. Moreover, Qiang et al. found that co-culture of NK cells with DMSCs resulted in downregulation of perforin, IFN-γ, and TNF-α, and upregulation of IL-4 and IL-10 in NK cells [88]. In addition, the interaction between M2 macrophages induced by MSCs and NK cells can inhibit the expression of proteins related to NK cell activation, such as NKp44, CD25, CD69, and IFN-γ. Other studies have shown that IFN-γ can enhance the immunomodulatory ability of MSCs. Considering the development time frame of adaptive immunity, if cell therapy is allowed to occur, NK cells may be the source of IFN-γ of MSCs in vivo [86].

## The immunomodulatory effects of MSCs combined with nerve conduits in the treatment of PNI

Some studies have suggested that stem cell transplantation combined with different nerve conduits may represent a promising strategy for the treatment of PNIs. Acellular nerve allografts (ANAs) are the most widely used biomaterials for nerve repair in the clinic. Recent studies have revealed that in the use of ANAs for bridging the nerve defects, T cells regulate the secretion of inflammatory cytokines within ANAs concerning the length of the ANA. For instance, long ANAs (4 cm) trigger a lower accumulation of T cells and cytokine levels of IFN-γ, IL-2, IL-4, and IL-13 [21]. Deng et al. found that T cells can regulate IL-4 by affecting eosinophils levels in ANAs [76]. ANAs combined with MSCs have been suggested to be effective for the treatment of PNI, and the regulation of the inflammatory microenvironment surrounding the injured nerve is currently being explored. For instance, Fan [89] et al. demonstrated that BMSCs combined with xenogeneic acellular nerve grafts (xANGs) could better promote nerve regeneration, possibly by reducing the release of pro-inflammatory factors IL-2, IFN-γ, and TNF-α and increasing the secretion of IL-10 in lymphocyte supernatants and serum when compared with those in the xANG group (5 mm). Yue et al used a 15 mm artificial nerve composed of epidermal neural crest stems cells (EPI-NCSCs), extracellular matrix (ECM), and polylactic co glycolic acid (PLGA) to treat peripheral nerve injury and found that after bridging with EPI-NCSCs, the expression of anti-inflammatory cytokines IL-4 and IL-13 increased, while the expression of pro-inflammatory cytokines IL-6 and TNF-α decreased [90]. Seven days after transplantation, M2 macrophages increased, while M1 macrophages decreased. Moreover, the number of SCs promoting myelination increased significantly 21 days after transplantation, while the number of activated fibroblasts decreased, and the structural and functional recovery of those animals treated with EPI-NCSCs was significantly better than in the DMEM blank control group. Therefore, the authors concluded that the combination of ANA with MSCs provides an inflammatory microenvironment suitable for sciatic nerve repair.

## Immunomodulatory effects of MSC-derived extracellular vesicles in PNI

A large body of research suggests that the majority of MSC-derived immunosuppressive effects are attributed to the immunoregulatory properties of the MSC-derived secretome, which is composed of a soluble component and encapsulated extracellular vesicles (EVs): apoptotic bodies, exosomes (exos), and microvesicles [91]. Due to their low immunogenicity, EVs derived from MSC (MSC-EVs) are considered as an appealing cell-free therapy. Recent studies have revealed MSC-EVs may exert anti-inflammatory functions that are similar to MSCs, and achieve their anti-inflammatory effects by reducing levels of inflammatory cytokines and enhancing anti-inflammatory responses [92]. Ma et al. found that hUCMSC-EVs can inhibit the expression of pro-inflammatory cytokines IL-6 and IL‐1β and up-regulate IL-10 expression to rebalance the inflammatory responses in a rat sciatic nerve injury model [93]. Sheng [94] et al. reported that rats treated with MSCs-EV and miRNA-22-loaded MSCs-EV (EV-miRNA-22) exhibited attenuated levels of pro-inflammatory factors TNF-α, IL-1β, and IL-18, and both showed a good ability to promote nerve regeneration, albeit the effect of EV-miRNA-22 was better than that of the EV group. On the other hand, miRNA-22 could inhibit the production and release of inflammatory responses. In addition, MSC-EVs can also exert immunomodulatory effects by modulating immune cells. For example, MSC-derived small extracellular vesicles (sEVs) can enhance IL-10 and TGF-β expression by inhibiting T lymphocyte proliferation and promoting their apoptosis, while decreasing the proportion of Th17 cells and increasing Treg cells in the spleen, leading to a decrease of IL-17 levels in serum [95]. Extracellular vesicles derived from CD73 modified hUC-MSCs can promote M2 macrophage polarization while decreasing the expression of pro-inflammatory factors TNF-α, IL-1β, and IL-6, and increasing the expression of anti-inflammatory factors IL-4 and IL-10 [96].

The immunomodulatory activity of MSC-Derived EVs may be regulated by Hypoxia-inducible factor 1-alpha (HIF-1α) [97]. Over-expression of HIF-1α in MSCs can enhance their immunosuppressive ability in different immune cell populations including DCs, monocytes, and NK cells [98]. Table 1 demonstrates the immunomodulatory effects of MSC-EVs for the attenuation of inflammatory diseases [80, 95, 99–128] (Table 1).

**Table 1:** Immunomodulatory effects of MSC/MSC-EVs for the attenuation of inflammatory diseases

Disease model	Animal for In Vivo study	MSC source	Effects on immune cells	Effects on cytokines	Signaling pathway/related exosomal cargo	Ref. No
Arthritis	In vitro	BMSCs,BMSCs-Exos	CD8 or CD4 T lymphocytes↓, B lymphocytes↑	IFN-γ,TNF-α↓; IL-10↑	–	[99]
IBD	Mouse	OE-MSCs-Exos	the differentiation of Th1 and Th17 cells↓, Treg cells↑	IL-17, IFN-γ↓; TGF-β, IL-10↑	–	[100]
		hP-MSCs-EVs	–	IL-10, TGF-β↑; TNF-α, IL-1β, IFN-γ and IL-6 ↓	–	[101]
		MuSCs	M2 macrophages↑,macrophages infiltration↓	IL-6,IL-1β↓	–	[102]
IUAs	Rat	UC-MSC-derived exosomes	M2 macrophage↑	IL-1β, IL-6 and TNF-α↓; IL-10, TGF-β↑	–	[103]
Liver disease	Mouse	hUC-MSCs	CD4 and CD8 T cells↓	IFN-γ↓	–	[104]
ALI	Mouse	UC-MSCs	M2 macrophage↑ M1 macrophage↓	TNF-α, IL-1β and IL-6↓	–	[105]
EAE	Mouse	BMSCs	T cells↓	–	STAT1,STAT3,mTOR	[106]
		hUCMSC-EV	the leukocyte infiltration↓,Tregs↑	IL-17a, TNF-α, and IFN-γ↓; IL-4, IL-10↑	–	[107]
Lung injury and fibrosis	Mouse, monkeys	hESCs	–	TNF-α, TGF-β1, IL-6, IL-1β, GRO-α, IL-1α, IL-3 and IL-8↓	–	[108]
Wound healing	Mouse	BMSCs	M2 polarization↑	IL-10↑; TNF-α↓	miR-223	[109]
Skin defect	Mouse	hBMSCs	M2 macrophages↑	–	miR-150-5p	[110]
IVD	Bovine	hBMSCs	–	IL-6, IL-8 and TNF-α ↓	–	[111]
Renal injury	Mouse	BMSC-exos	infiltration of of macrophages↓	TNF-α, IL-6 and IL-1β↓	CCL2	[112]
Renal Diseases	Rat	hUCMSC-Ex	–	TNF-α, IL-6 and IL-1β↓	mTOR	[113]
cGVHD	Mouse	BMSC-exos	Th17 cells, CD4 + T cells↓, Tregs↑	IL-17, IL-17a, IL-21, IL-22 and IL-2↓; IL-10↑	–	[114]
aGVHD	Mouse	BMSC-exos	CD8αDCs, CD11b cDCs↑; CD8, CD3CD4 T cells↓	IL-2, TNF-α and IFN-γ↓; IL-10↑	–	[115]
Heart allograft rejection	Rat	IDO-BMSCs	Tregs↑, DC↓	IL-10, TGF-β1, TGF-β2 and TGF-β3↑; IL-2, IFN-γ↓	–	[116]
CIA	Rat	hUCMSCs	T lymphocytes (proliferation↓, apoptosis ↑), Th17 cell↓, Tregs↑	IL-17, TGF-β↓	RORγt mRNA,Foxp3 mRNA	[117]
	Mouse	hADMSCs	Tregs↑,the proliferation of T cells↓	TNF-α, IL-1β and IL-6↓; IL-10↑	–	[80]
	Rat	hUCMSC-sEVs	Th17 cell↓, T lymphocyte proliferation↓, Tregs↑	IL-17↓; IL-10, TGF-β ↑	–	[95]
PNI	Rat(MRI, LPS and FK506)	GFP-BMSCs	M1,TLR4↓	TNF-α, IL-6↓; BDNF, GDNF↑	–	[118]
PSNL	Rat(LPS)	BMSCs	–	IL-1β, TNF-α↓	–	[119]
CCI	Rat(PMF)	ADMSCs	–	IL-6, IL-1β↓; IL-10↑	–	[120]
Diabetic	Rat	DPSCs	CD206↑, CD68‐positive monocytes/macrophages↓	TNF-α, IL-1β↓; IL-10↑	–	[121]
CCI and SNI	Mouse(TGF-β1)	BMSCS	–	Il-1β, IL-6 and TNF-α↓; TGF-β1↑	–	[31]
Nerve injury-induced pain	Rat	UCMSC -exos	–	TNF-α, IL-1β↓	–	[122]
Dysphagia	Rat	hDPSCs	M2 macrophages↑	iNOS, IL-1β↓	–	[123]
TBI	Rat	MSCs	M1 macrophage↓, M2 macrophages↑	IL-10↑; IL-1β, IL-6, IL-17, IFN-γ and TNF-α↓	NF-κB	[124]
DAH	Mouse	hUCMSC-Ex	M1 macrophage↓, M2 macrophages↑	IL-6, TNF-α↓; IL-10, TGF-β↑	–	[125]
Skeletal muscle contusion	Mouse and in vitro	hBMSC-exos	M1 macrophages ↓, M2 macrophages↑	IL-6, TNF-α↓; IL-10,TGF-β↑	–	[126]
SCI	Mouse	hUCMSCs	M2 macrophages↑	IL-7, IFN-γ, and TNF-α↓; IL-4, IL-13↑	–	[127]
PF	Mouse	hUCMSCs	Tregs↑	CXCL9, CXCL10↑	–	[128]

Effect of MSCs on cytokines and immune cells when treating different diseases. BMSCs: bone marrow-derived mesenchymal stem cells; OE-MSCs: Olfactory ecto-mesenchymal stem cells; IBD: inflammatory bowel disease; IUAs: intrauterine adhesions; UC-MSC: umbilical cord-derived mesenchymal stem cell; ALI: acute lung injury; EAE: experimental autoimmune encephalomyelitis; hESCs: human embryonic stem cells; hP-MSCs-EVs: human placental mesenchymal stem cells-derived EVs; hUCMSC-Ex: human umbilical cord MSC-derived exosomes; cGVHD: chronic Graft-Versus-Host Disease; aGVHD: acute graft-versus-host disease; cDCs: the CD8α conventional dendritic cells; IDO-BMSCs: BMSCs which stably expressed IDO; CIA: collagen-induced arthritis; NCI: nerve crush injury: MRI: magnetic resonance imaging; PSNL: partial sciatic nerve ligation; CCI: chronic constriction nerve injury model; ADMSC: adipose tissue derived mesenchymal stem cells; PMF: pulsed magnetic field; DPSCs: Dental pulp-derived stem cells; SNI: spared nerve injury; TBI: traumatic brain injury; DAH: diffuse alveolar hemorrhage; MuSCs: muscle stem cells; SCI: spinal cord injury; hUCSC-EV: Human umbilical cord mesenchymal stem cell-derived extracellular vesicles; EAE: experimental autoimmune encephalomyelitis; PF: Pulmonary fibrosis; RA: Rheumatoid arthritis; hUCMSC-sEVs: hUCMSC-derived small extracellular vesicles (sEVs)

## Conclusion and deficiency

MSCs are multipotent stem cells with multiple biological potentials and may represent an ideal option for cell therapy applications due to their regenerative and immunoregulatory functions [129]. The use of MSCs with innate mechanistic features to mediate the local inflammatory response following PNI would both reduce loss of muscle mass and shift the microenvironment towards a pro-regenerative rather than profibrotic phenotype [130]. Animal studies with intravenous reinfusion transplantation or local tissue injection of MSCs have found that these cells can quickly migrate to injury sites and localize to sites of information where they promote anti-inflammatory and immune regulatory effects [131–133]. There is a correlation between higher MSCs survival and reduced levels of pro-inflammatory cytokines as well as a transformation in macrophages from M1 to M2 [134]. However, currently studies investigating the immunoregulatory mechanisms of MSCs for the treatment of PNI are not comprehensive enough, and the functions played by different immune cells and cytokines remain to be studied. Moreover, the various pathways involved in the mediation of MSC-derived benefits remain to be explored. Stem cell transplantation studies are at this point predominantly in pre-clinical stages, with several issues needing to be addressed. Although the effectiveness of MSCs/MSCs-EVs for the treatment of PNI has been demonstrated many times in animal studies, there are still few studies highlighting the exact therapeutic mechanisms, in particular relating to the immunoregulatory mechanisms exerted by MSCs/MSCs-EVs. Further studies investigating the molecular mechanism underlying the beneficial effects of MSCs in the treatment of PNI will provide new strategies for disease therapy.

## Data Availability

Not applicable.

## Abbreviations

PNI: Peripheral nerve injuries
MSCs: Mesenchymal stem cells
TNF-α: Tumor necrosis factor-α
IL: Interleukin
IFN: Interferon
TGF: Transforming growth factor
HO: Heme oxygenase
HGF: Hepatocyte growth factor
NO: Nitricoxide
HLA-G5: Human leucocyte antigen-G5
PGE: Prostaglandin E
NK: Natural killer
DCs: Dendritic cells
exos: Exosomes
SCs: Schwann cells
LN: Lymph nodes
TLR: Toll like receptor
Treg cells: Regulatory T cells
GDNF: Glial cell derived neurotrophic factor
CXCL: Cysteine-X-cysteine chemokine ligand
CCL: Cellular components chemokine ligand
BMSCs: Bone marrow-derived mesenchymal stem cells
ADMSCs: Adipose tissue derived mesenchymal stem cells
GM-CSF: Granulocyte–macrophage colony-stimulating factor
NF-κB: Nuclear factor-κB
SCI: Spinal cord injury
Teffs: Effector T cells
WJ: Wharton’s jelly
DP: Dental pulp
PBMCs: Peripheral blood monocytes
TSG-6: Tumor-necrosis-factor-inducible gene 6 protein
PR: Progesterone receptor
GR: Glucocorticoid receptors
hUC-MSCs: Human umbilical cord-derived mesenchymal stem cells
TBI: Traumatic brain injuries
COX: Cyclooxygenase
ANAs: Acellular nerve allografts
xANGs: Xenogeneic acellular nerve grafts
EPI-NCSCs: Epidermal neural crest stems cells
ECM: Extracellular matrix
PLGA: Polylactic co glycolic acid
EVs: Extracellular vesicles
OE-MSCs: Olfactory ecto-MSCs
IBD: Inflammatory bowel disease
IUAs: Intrauterine adhesions
ALI: Acute lung injury
EAE: Experimental autoimmune encephalomyelitis
hESCs: Human embryonic stem cells
hP-MSCs-EVs: Human placental mesenchymal stem cells-derived EVs
hUCMSC-Ex: Human umbilical cord MSC-derived exosomes
mTOR: Mammalian target of rapamycin
cGVHD: Chronic Graft-Versus-Host Disease
aGVHD: Acute graft-versus-host disease
cDCs: The CD8α conventional dendritic cells
IDO-BMSCs: BMSCs which stably expressed IDO
CIA: Collagen-induced arthritis
NCI: Nerve crush injury
MRI: Magnetic resonance imaging
Foxp3: Forkhead box protein P 3
PSNL: Partial sciatic nerve ligation
CCI: Chronic constriction nerve injury model
PMF: Pulsed magnetic field
DPSCs: Dental pulp-derived stem cells
SNI: Spared nerve injury
TBI: Traumatic brain injury
DAH: Diffuse alveolar hemorrhage
MuSCs: Muscle stem cells
hUCSC-EV: Human umbilical cord mesenchymal stem cell-derived extracellular vesicles
PF: Pulmonary fibrosis
RA: Rheumatoid arthritis
